# “Like One Long Battle:” Employee Perspectives of the Simultaneous Impact of COVID-19 and an Electronic Health Record Transition

**DOI:** 10.1007/s11606-023-08284-3

**Published:** 2023-10-05

**Authors:** Justin M. Rucci, Sherry Ball, Julian Brunner, Megan Moldestad, Sarah L. Cutrona, George Sayre, Seppo Rinne

**Affiliations:** 1 Center for Healthcare Organization and Implementation Research, Boston, VA USA; 2 The Pulmonary Center, Department of Medicine, Boston University, Boston, MA USA; 3 VA Northeast Ohio Healthcare System, Cleveland, OH USA; 4 Center for the Study of Healthcare Innovation Implementation and Policy, VA Greater Los Angeles Healthcare System, Los Angeles, CA USA; 5 Seattle-Denver Center of Innovation for Veteran-Centered and Value-Driven Care, VHA Puget Sound Health Care System, Seattle, WA USA; 6 Center for Healthcare Organization and Implementation Research, Bedford, VA USA; 7 Department of Population and Quantitative Health Sciences, UMass Chan Medical School, Worcester, MA USA; 8 University of Washington School of Public Health, Seattle, Washington USA

**Keywords:** electronic health records, COVID-19 pandemic, burnout, informatics, organizational change

## Abstract

**Background:**

Healthcare organizations regularly manage external stressors that threaten patient care, but experiences handling concurrent stressors are not well characterized.

**Objective:**

To evaluate the experience of Veterans Affairs (VA) clinicians and staff navigating simultaneous organizational stressors—an electronic health record (EHR) transition and the COVID-19 pandemic—and identify potential strategies to optimize management of co-occurring stressors.

**Design:**

Qualitative case study describing employee experiences at VA’s initial EHR transition site.

**Participants:**

Clinicians, nurses, allied health professionals, and local leaders at VA’s initial EHR transition site.

**Approach:**

We collected longitudinal qualitative interview data between July 2020 and November 2021 once before and 2–4 times after the date on which the health system transitioned; this timing corresponded with local surges of COVID-19 cases. Interviewers conducted coding and analysis of interview transcripts. For this study, we focused on quotes related to COVID-19 and performed content analysis to describe recurring themes describing the simultaneous impact of COVID-19 and an EHR transition.

**Key Results:**

We identified five themes related to participants’ experiences: (1) efforts to mitigate COVID-19 transmission led to insufficient access to EHR training and support, (2) clinical practice changes in response to the pandemic impacted EHR workflows in unexpected ways, (3) lack of clear communication and inconsistent enforcement of COVID-19 policies intensified pre-existing frustrations with the EHR, (4) managing concurrent organizational stressors increased work dissatisfaction and feelings of burnout, and (5) participants had limited bandwidth to manage competing demands that arose from concurrent organizational stressors.

**Conclusion:**

The expected challenges of an EHR transition were compounded by co-occurrence of the COVID-19 pandemic, which had negative impacts on clinician experience and patient care. During simultaneous organizational stressors, health care facilities should be prepared to address the complex interplay of two stressors on employee experience.

**Supplementary Information:**

The online version contains supplementary material available at 10.1007/s11606-023-08284-3.

## INTRODUCTION

Healthcare systems regularly navigate a variety of external stressors, including policy changes, natural disasters, workplace violence committed by patients or other community members, and emerging infectious diseases^1–4^. These phenomena threaten usual delivery of patient care, and while strategies exist to anticipate and manage these challenges^5^, there is a lack of data regarding how health care systems experience and address concurrent organizational stressors.

Starting in late 2020, a Department of Veterans Affairs (VA) medical center experienced two markedly disruptive events—a transition to a new electronic health record (EHR) and a surge in COVID-19 cases. Transitions from one EHR to another are increasingly common^6^. These undertakings can introduce challenges to patient care as clinicians and staff must unlearn existing technologies and adapt to new roles and workflows^6^. The VA is the largest integrated healthcare system in the USA, and it is currently undergoing a national conversion from its legacy EHR to Oracle Cerner (“Cerner”) that is expected to last at least 10 years and cost more than 39 billion dollars^7^. This represents one of the largest EHR transitions in history; the large scope of this undertaking could amplify the challenges identified in previous transitions.

The COVID-19 pandemic also had wide-ranging impacts on patient care. During surges of COVID-19 cases, hospital systems required increased critical care resources and sought out novel care delivery practices^8,9^. Simultaneously, there was delayed or foregone outpatient care for medical conditions unrelated to COVID-19^10–12^. Facilities rapidly enacted sweeping systemic changes in response to the pandemic. Telemedicine platforms were widely adopted to continue providing care while mitigating COVID-19 transmission^13,14^, common pre-pandemic medical practices were re-assessed to maximize healthcare resources^15^, and the medical trainee experience was adjusted through virtual educational activities and additional reimbursement^16,17^. Despite all that was learned from these attempts to mitigate the systemic impact of COVID-19, evidence regarding best practices to navigate simultaneous organizational stressors to healthcare delivery remains limited.

We sought to understand how front-line clinicians and staff experienced concurrent organizational stressors during VA’s EHR transition amid the COVID-19 pandemic. Evaluating this experience could identify strategies to effectively manage future EHR transitions that coincide with unanticipated external factors and may provide broader insight into navigating simultaneous organizational stressors.

## METHODS

### Study Design

We are a multidisciplinary team of investigators and clinicians that has been conducting a multi-site, mixed-methods evaluation of clinician and staff experience with the VA EHR transition. In this longitudinal undertaking, findings from initial sites have been used to enhance operational procedures at subsequent VA transition sites, and to further inform our ongoing assessment of the transition. The current study is a secondary analysis of qualitative data collected from the first VA medical center to begin using the new EHR﻿, and seeks to describe the experience of simultaneously navigating the EHR transition and a surge of COVID-19 cases.

This quality improvement project was designated as non-research by the VA Bedford Healthcare System Institutional Review Board.

### Site Description

**Figure 1:**
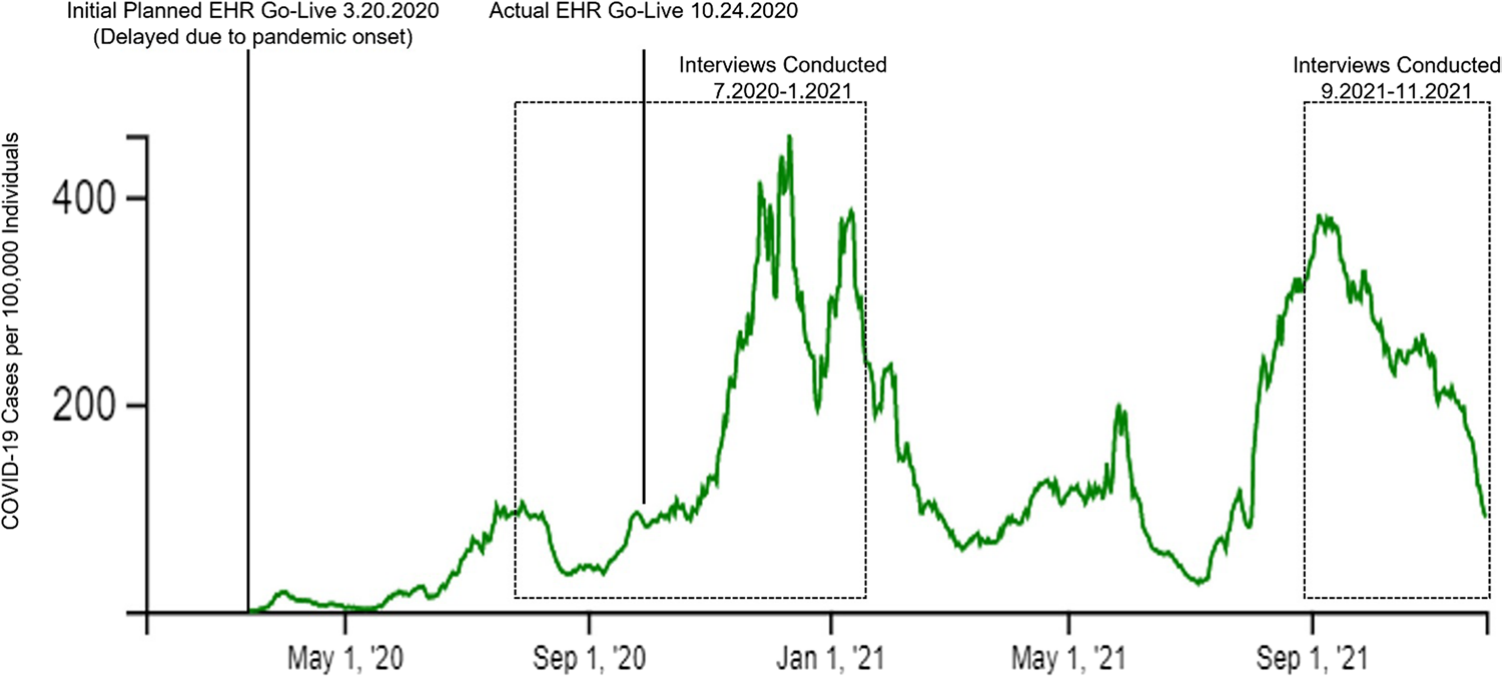
Timeline of an electronic health record (EHR) transition at a Veterans Affairs Medical Center, and the corresponding local trends in COVID-19 cases. Vertical lines and dashed boxes designate key events in the qualitative evaluation of the EHR transition. The green line represents the weekly case rate of COVID-19 per 100,000 individuals in the county where the healthcare facility is located^40^

The first VA facility to undergo the EHR transition is comprised of a network of inpatient and outpatient sites that cares for more than 30,000 veterans annually^18^. It includes a main campus with 36 inpatient hospital beds, 34 rehabilitation-oriented nursing home beds, and an array of primary care, behavioral health, and medical specialty services. Separately, the healthcare system has six community sites that provide additional primary and specialty outpatient care^19^. The facility transitioned to Cerner on October 24th, 2020—a date subsequently referred to as “go-live”—which coincided with the beginning of a local surge of COVID-19 cases (Fig. 1).

### Participants

We identified participants through direct outreach and snowball sampling. In preliminary meetings with local leaders and key stakeholders at the initial transition site, we identified clinical teams with potential interest in participating. We then spoke with team leaders to obtain their buy-in and identify team member participants. The final group of participants included local clinical leaders and clinicians (physicians, clinical pharmacists, and psychologists [*n*=16 total]), nurses (registered nurses and licensed practical nurses [*n*=6 total]), and allied health professionals (*n*=8 total) (Table 1). Four participants were “Cerner champions,” who were dedicated to supporting and advocating for the EHR transition and seeking to overcome organizational resistance^20^.

**Table 1:** Participant roles and data collection time-points

	Pre Go-Live		Post Go-Live			Total
	Stakeholder engagement meetings	Pre-go-live interviews	Check-ins	2 months post-go-live interviews	10 months post-go-live interviews	
Clinicians* and clinical leaders (*n*=16)	11	11	22	14	13	**71**
Nurses^†^ (*n*=6)	0	6	12	4	5	**27**
Allied health professionals^‡^ (*n*=8)	0	4	13	5	2	**24**
Total	**11**	**21**	**47**	**23**	**20**	**122**

*Physicians, clinical pharmacists, psychologists, advanced practice nurses

^†^Registered nurses and licensed practical nurses

^‡^Medical assistants, phlebotomists, counselors, audiologists, physical therapists

Bolded numbers represent total values across rows and columns

### Data Collection

To capture the breadth of experience at the initial EHR transition site, we completed semi-structured interviews and check-ins with each participant at multiple time-points relative to go-live. This included stakeholder engagement meetings, pre-go-live interviews, immediately post-go-live interviews (brief biweekly check-ins and full interview at 2–3 months after go-live), and 10-month post-go-live interviews (Table 1). Immediately post-go-live interviews were brief check-ins (approximately 15–20 min) to minimize burden on participants, while all other interviews lasted approximately 60 min. The interview timeline overlapped with local surges in COVID-19 cases (Fig. 1). Interviews were conducted via phone or Microsoft Teams^®^ by nine team members with experience in qualitative research. Interviews were audio-recorded and professionally transcribed verbatim. Interview guides were designed by our qualitative research team to elicit information across six main topic areas that informed and improved implementation of the new EHR, including attitudes toward Cerner, information, preparation, training and education, resources, and prior experience with EHRs and EHR transitions (interview guides are available in Supplementary Appendix 1).

### Analysis

We used a combination of deductive and inductive content analysis to evaluate interview transcripts^21^. We first created a collection of categories derived from a priori defined topics of interest related to the experience of the VA EHR transition. Next, we conducted line-by-line coding of transcripts using qualitative analysis software (ATLAS.ti, version 9), and organized codes within the categories. New codes and categories were added when concepts emerged that did not align with the pre-defined classification.

During the analytic process, “COVID” emerged as an inductive category that we sought to explore more deeply. This category included 18 total codes across a range of topics, including, but not limited to “COVID-19 delay,” “COVID-19 impact,” “more f/u care due to COVID,” etc. For this secondary analysis, we reviewed all codes and their associated quotes within the COVID category. We separately performed keyword searches for “COVID,” “COVID-19,” and “pandemic” across all transcripts, to ensure all appropriate quotes were captured. We used content analysis and group discussion^22^ to identify prevailing themes that described the experiences of clinicians and staff who simultaneously navigated the COVID-19 pandemic and the EHR transition. We selected representative quotes through group consensus at team meetings that were felt to be most emblematic of the identified themes. We added boldface to emphasize key messages.

## RESULTS

We conducted 122 interviews with 30 unique participants; there was variable participation across interview and “check-in” time points (Supplementary Table 1). In general, participants described multiple challenges in navigating the EHR transition and concurrent surges of COVID-19 cases across interview time-points. We identified five themes that encompassed the front-line experience: (1) efforts to mitigate COVID-19 transmission led to insufficient access to EHR training and support, (2) clinical practice changes in response to the pandemic impacted EHR workflows in unexpected ways, (3) lack of clear communication and inconsistent enforcement of COVID-19 policies intensified pre-existing frustrations with the EHR, (4) managing concurrent organizational stressors increased work dissatisfaction and feelings of burnout, and (5) participants had limited bandwidth to manage competing demands that arose from concurrent organizational stressors.

### Efforts to Mitigate COVID-19 Transmission Led to Insufficient Access to EHR Training and Support

In response to the COVID-19 pandemic, the initial VA transition site designed policies to protect the health and safety of VA staff such as promoting telework where possible, masking and physical distancing of in-person employees, and isolation of individuals who tested positive for COVID-19. These strategies, though necessary and appropriate for management of the pandemic, negatively impacted EHR training:

“I worked at home for 6 months, and since **I was working from home** and couldn’t go <…> for the in-person training, then **it was, well you can’t be a superuser then**, hey, **we need to pick someone else** and throw them into the fire.” (Allied health professional, 10 months post)

“I think **it’s dragging out some of the training** because they can’t fit hardly anybody in the room anymore. **It used to be they’d have 4 computers in a row, and now they’ve got 2, to try and space us out.**” (Nurse, pre)

At the same time, efforts to mitigate transmission of COVID-19 also limited in-person availability of Cerner staff who were supposed to provide real-time support throughout the EHR transition. These challenges were compounded by uncertainty among VA clinicians and staff as to how to access virtual support from Cerner staff:

“Because of COVID, **a lot of the Cerner people** who were here to help, they **have to telework.”** (Allied Health Professional, check-in 2)

“My understanding is that a bunch of our elbow support from the **Cerner folks** <…> **were sent home because of too many COVID cases** <…> But essentially, they were sent home like a week early and are just doing virtual support I guess**, I have no idea how to even get that virtual support**.” (Clinician, check-in 1)

### Clinical Practice Changes in Response to the Pandemic Impacted EHR Workflows in Unexpected Ways

Participants described how changes to their day-to-day clinical activities driven by the pandemic led to unanticipated challenges working within the new EHR. For example, unexpected scheduling changes during the pandemic were difficult to monitor and manage in the new EHR system:

“And the other thing I’ve noticed at least is how we schedule our clinics and how clinics are built. The COVID-19 guidance, **there’s different ways our clinics have been built, and that hasn’t necessarily transferred over**.” (Allied health professional, pre)

“With the way we are with the pandemic, on a weekly basis the providers have a face-to-face clinic one day a week. And they also have a phone call clinic, and a video clinic**. So, we have 3 clinics, per provider**, minimum, **that you have to be constantly looking at**, are they scheduled in the proper way?” (Nurse, pre)

Similarly, fewer warm handoffs were occurring as providers tried to limit face-to-face interactions. This change in usual practice, combined with challenges placing referrals within the new EHR, decreased the effectiveness of communication among team members and had the potential to limit access to mental health care for patients.

“**Before Cerner we [mental health providers] were really functioning as intended** <…> that **patients have access to a mental health provider the same day they see their primary care provider**. It really increases the likelihood that they’re actually going to engage in care <…> **But with COVID and Cerner, we really haven’t been functioning on that model** <…> So **instead of getting more of those same day warm handoff contacts**, those are really being reserved for the folks who maybe have more urgency or higher distress levels, and **the rest are going to referrals** <…> **And the referral pathway** to [the mental health team] **wasn’t working for a really long time.”** (Clinician, 10 months post)

Many individuals were forced to take on new or different roles because of the pandemic. Adapting to these new roles was made more difficult by the need to learn additional functions within the EHR, and affected functioning of the team from which individuals were pulled:

“And now we’re in surge mode, and **they’re pulling primary care providers, and they’re putting them in the hospital as hospitalists**. And, you know that’s scary, because <…> **we’re all going to have to learn Cerner in the inpatient capacity**.” (Clinician, 2 months post)

“So **other team members had to pick up the job that I would normally be doing**, even for phone calls, and pre-calls, and preplanning. And that happened with probably the majority of primary care, that we were just pulled every which way we could, for those that were detailed to other areas. **So that had a big impact** on primary care.” (Nurse, pre)

“**We have to have a screener at the front door every day** to do temperatures, ask the questions <…> ensure they’re masked. And **we have to pull from staff [for that position**]. Again, **that affects time on documentation or helping others, or being in class for Cerner**.” (Nurse, pre)

Like many health care systems, VA greatly expanded its telehealth infrastructure to continue providing care for patients while minimizing the risk of COVID-19 transmission. Many participants noted the hardships of undergoing an EHR transition while simultaneously adjusting to a new patient-care approach:

“Because now **we are supposed to transfer everybody over to telehealth** unless they insist on being seen face-to-face. **And then we have Cerner**.” (Clinician, 2 months post)

“But what happens is then **you’re doing a whole lot of work trying to support them on the outside without being able to have them come in. So that actually requires more work** than it does slipping them in an office visit, taking a look at their knee, taking care of it, and then they’re gone.” (Clinician, pre)

In contrast to these criticisms, some participants did identify a silver lining to the alignment of pandemic surges and the EHR transition. The mandated decrease in patient volume to mitigate COVID-19 transmission allowed more time to navigate the new EHR:

“**With COVID, now we’re still supposed to be at reduced,** 25% or below **capacity**. <…> **So, in that way, it kind of worked out towards our favor**, because <…> due to COVID﻿ ﻿we can’t have as many [patients].” (Nurse, 2 months post)

### Lack of Clear Communication and Inconsistent Enforcement of COVID-19 Policies Intensified Pre-existing Frustrations with the EHR

Participants described increasing frustration and conflict within their local teams and the wider VA institution while coping with the pandemic and the EHR transition. There was a notable desire for improved communication from VA leaders. For example, one participant was dissatisfied with the lack of clarity surrounding the expected volume of in-person patient visits as the number of COVID-19 cases increased:

“Because **we’re kind of getting geared up for the surge** <…> **at the same time we’re dealing with Cerner** <…> So they’re dialing it [number of in-person office visits] back, but when I asked the director during the meeting on Thursday, **when are we dialing it back? What are we dialing it back to? And when does it start? And he was non-committal**, he just said, ‘you have to do what you think is right.’” (Clinician, check-in 2)

Additionally, participants were frustrated with the short notice regarding plans to relocate providers to locations that needed more support for COVID-19-related patient care. The limited timeframe to prepare for these changes exacerbated ongoing challenges managing the EHR transition:

“So they’ve already sent emails out to nurses that they will more likely than not be getting detailed to inpatient services. This is what happened in the spring, and the **providers were given less than 24-hour notice that they were going to be inpatient**. This went out yesterday, it’s starting today.” (Clinician, check-in 2)

Individuals demonstrated differences in commitment to masking, at least partially due to poor communication surrounding masking policy from leadership and lack of effective policy enforcement. While respondents did not directly describe the interaction between masking and the EHR transition, they noted that variable masking practices negatively affected team dynamics, which could substantially hinder the EHR transition.

**“[Working with others during the EHR transition is] complicated** some by COVID and **the lack of clear guidance on masking**, because **our primary care people haven’t been masking** regularly. And **they don’t seem to make them**.” (Clinician, 2 months post)

“It’s the nursing staff, it’s the MSAs [Medical Support Assistants], it’s the cleaning staff, some of the cleaning staff. Some of them are wearing masks, some of them don’t. You know. **And I confronted them one night when I was there late <…> because I said, ‘put your mask on** buddy, I don’t want you dying of this thing’ <…> **And he said, ‘most of the places I work, there’s not other people around.’ And I’m like, ‘well I’m here, what am I, chopped liver?’”** (Clinician, check-in 3)

### Managing Concurrent Organizational Stressors Increased Work Dissatisfaction and Feelings of Burnout

The overlapping burdens of COVID-19 and the EHR transitions elicited intense emotional responses among many participants. Some described maximal levels of stress among clinicians and staff:

“But you know, **everyone is already stressed to the max** because you’re in the middle of a pandemic, and then you’re going to throw new software on it.” (Allied health professional, 2 months post)

Others seemed to experience a notable loss of resiliency after co-managing the pandemic and a new EHR that they felt was not performing to meet their needs:

“**The entire facility is overwhelmed, worn out. It’s been like one long battle**. We are all so exhausted, and you know, [Cerner] came with COVID, so we can’t negate the impact of that, but just having a system that we seriously don’t feel works very well.” (Clinical leader, 10 months post)

Low morale seemed to be a common thread among participants, who noted difficulties keeping a positive outlook in the face of concurrent stressors:

“And that’s just because I think of everything going on, **being in the middle of a pandemic**, our COVID cases around the area are increasing. We’re having COVID positive within the hospital. **And then now software I think is just pushing everyone a little too much** right now.” (Allied health professional, Check-in 3)

“Part of it is, **it’s exhausting to talk through a mask** to hearing impaired people all of the time. And **then you add Cerner in there**. It’s just, **morale is low**.” (Clinician, check-in 3)

“I liked my job, you know. And **now I’m not liking my job** <…> **there’s Cerner, there’s COVID, those two things together** make it challenging.” (Clinician, check-in 4)

### Limited Bandwidth to Manage Competing Demands

There was a pervasive feeling among participants of difficulty managing the demands of multiple simultaneous changes:

“It’s basically**, we’re changing multiple factors at once** where the acuity is going up with our veterans, and their needs are going up, and at the same time we’re struggling with the charting component <…> **it’s kind of two things working against us at once**.” (Nurse, check-in 3)

“**We haven’t healed, we haven’t even gotten through this horror show, and now there’s another horror show**. So, to call it bad timing to implement this is an understatement.” (Clinician, 2 months post)

Participants voiced the belief that the challenges of competing demands could have been avoided by deferring the EHR transition in the face of the obvious burdens of the pandemic:

“So, we’re dealing with all of this [the EHR transition] <…> in a pandemic. **It might’ve been a little easier if they wouldn’t have launched this in the midst of a pandemic**”. (Allied health professional, check-in 4)

“Every single bit of **this was absolutely predictable and avoidable**. All they had to do was wait, fix this, **let this pandemic do what it’s going to do, and hold off on Cerner**. That’s what should’ve happened.” (Clinician, 2 months post)

One participant summarized the co-occurrence of pandemic surges and the EHR transition in terms of a force of nature:

“And then at the same time [as the EHR transition] you have a surge [of COVID-19 cases] <…> I mean, **you couldn’t ask for a worse storm**.” (Clinician, 10 months post)

## DISCUSSION

In this qualitative study, we describe the front-line perspectives of VA clinicians, nurses, and allied health professionals who were simultaneously navigating an EHR transition and the COVID-19 pandemic. Attempts to mitigate one stressor often had unintended consequences that affected management of the other; participants struggled to anticipate and reconcile these multidimensional interactions. Participant frustrations were exacerbated by lack of clear communication from leadership, and ultimately culminated in decreased employee work satisfaction and increased feelings of burnout. Given the expected rise in future EHR transitions^6^, the occurrence of an EHR-to-EHR transition during a healthcare crisis offers much-needed insight to other facilities planning EHR transitions, and more broadly provides a valuable description of how healthcare systems experience concurrent organizational stressors.

Responding to COVID-19 resulted in unexpected challenges for the EHR transition. Complex sociotechnical systems can impact employees in unpredictable ways^23^; participants described how these separate stressors on the healthcare system interacted, hindering efficient delivery of patient care. For example, the need to decrease COVID-19 transmission by promoting remote work and quarantining staff after COVID-19 exposures led to insufficient access to EHR training and support. Additionally, interventions to manage patient care during the pandemic—increasingly complex scheduling, changes in usual referral workflows, and evolving clinical roles—exacerbated adoption of a new EHR system. On rare occasions, the pandemic had unexpected positive effects, such as when decreased volume of in-person office visits provided additional time to chart within the new EHR.

While the COVID-19 pandemic was a unique stressor that necessitated targeted interventions, the organizational response to COVID-19 yielded numerous valuable lessons that have informed policies and hospital preparations for other organizational stressors, including future pandemics and natural disasters^24–26^. Our study adds to these lessons learned by offering insight into how organizations may navigate co-occurring stressors. Understanding how to mitigate co-occurring stressors is critical for healthcare organizations to prepare adequately for future EHR transitions and other organizational changes. For example, unplanned healthcare policy changes occurring during an EHR transition could drive new patient care workflows that strain EHR adoption. Increasingly common natural disasters, known to increase hospital admissions^27^ and impact supply chains, could further exacerbate care delivery disruptions already occurring during a transition.

There are few studies evaluating concurrent emergency management in healthcare settings^28–30^. Available findings from the limited literature highlight the importance of proactive (as opposed to reactive) strategies to combat the combined impact of dual stressors whenever possible^31^. Anticipated disruptions to care, such as EHR transitions, are likely more amenable to proactive planning for resilience in the face of a second external stressor. Adapting a dynamic sustainability framework may provide a structure to minimize the compounded disruption to care when a second stressor occurs simultaneously with an EHR transition^32^. The dynamic sustainability framework suggests that allowing an intervention to evolve within a changing system improves sustainability and maximizes generalizability to other practice settings. By designing an EHR implementation strategy that remains flexible in the face of unanticipated challenges, healthcare facilities can optimize resiliency in the face of unexpected external stressors. For example, nimble training strategies allowing for asynchronous and virtual learning when barriers to in-person attendance occur, as well as malleable EHR workflows that can adapt to unexpected practice changes, may translate to improved experiences for frontline staff.

The negative feelings created by the convergence of the pandemic and EHR transition led to increasing work dissatisfaction, heightened interpersonal conflict, and feelings of burnout. Healthcare systems globally are grappling with the burnout crisis^33^, which is negatively impacting healthcare worker mental health, increasing turnover, contributing to lower patient care quality^34^, and significantly increasing cost to the health care system (estimated at more than $4 billion dollars annually in the USA)^35^. Despite increased recognition, managing burnout among health care professionals remains a challenge. Evidence-based strategies to mitigate burnout^34,36^ appear to be critical when an organization is managing concurrent stressors. Structural interventions such as changes in intensity of workload or improvements in care delivery typically have the strongest impact on burnout^36^. Unfortunately, in the midst of a pandemic that disrupted care delivery, imposed longer hours, and intensified workdays, these interventions may not have been feasible. The optimal strategies to prevent and manage healthcare worker burnout during a pandemic are incompletely understood, but evidence suggests that interventions to improve mental health must address the needs of frontline workers, remain flexible to local care settings, and use effective communication^37^.

Participants who were overstretched due to heightened demands of the co-occurring pandemic and EHR transition were further frustrated by poor communication and inconsistent policy enforcement from VA leaders. Lack of definitive timelines and limited notice prior to implementation of policy-changes caused distress. While clear, timely, and consistent messaging from healthcare leaders is always beneficial^38^, it appears to be vital during concurrent organizational stressors. Application of a transformational leadership approach^39^, in which leaders effectively communicate a universal vision that inspires employees toward a common goal, could be applied during future simultaneous stressors. If VA leaders had effectively conveyed empathy with the front-line staff transition experience, while simultaneously fostering a shared commitment to effectively support Veterans at a time of dire need (due to the co-occurrence of the pandemic), it is possible that participants may have rallied around this goal and reflected more positively on communication from leadership.

Our study has important strengths. It captured unique perspectives of individuals from across the spectrum of health care providers. The longitudinal data collection allowed us to describe the lived experience throughout the co-occurring COVID-19 pandemic and EHR transition, and to align participant experiences with the natural evolution of local pandemic surges.

There are potential limitations as well. Our findings came from 30 participants at one VA healthcare system, which may limit the generalizability of findings to other VA and non-VA healthcare systems undergoing EHR transitions. While we sought out diverse perspectives, participants all voluntarily took part in this study during a challenging period, which may have resulted in individuals with strong opinions regarding the EHR transition and/or the COVID-19 pandemic self-selecting for inclusion. We did not conduct analyses to assess for evolution of individual participant experiences over time, and this should be considered in future studies evaluating the EHR transition experience. Despite our multi-pronged approach to identify participant experience related to COVID-19 through use of codes and keyword searches, we may have missed some reverberating effects of the pandemic on the EHR transition that were not explicitly identified by participants as being impacted by the pandemic.

## CONCLUSION

The co-occurrence of the COVID-19 pandemic and an EHR-to-EHR transition was profoundly challenging for VA clinicians, nurses, and allied health professionals, who struggled to manage the competing demands of concurrent disruptive events. Our research offers insight into managing future co-occurring challenges and may inform planning and preparation for organizational change. When possible, healthcare systems should try to anticipate and avoid simultaneous stressors, although it is not always possible to delay necessary organizational initiatives, and unforeseen circumstances may arise. Future EHR transitions may be less impacted by external stressors if healthcare systems employ flexible implementation strategies within the context of a dynamic sustainability framework, design interventions to mitigate healthcare worker burnout, and optimize communication from leadership.

### Supplementary Information

ESM 1 (DOCX 42.9 kb)

## References

[1] Levine DM, Chalasani R, Linder JA, Landon BE (2022). Association of the Patient Protection and Affordable Care Act With Ambulatory Quality, Patient Experience, Utilization, and Cost, 2014-2016. JAMA Netw Open..

[2] Phillips JP (2016). Workplace Violence against Health Care Workers in the United States. N Engl J Med..

[3] Redlener I, Reilly MJ (2012). Lessons from Sandy--preparing health systems for future disasters. N Engl J Med..

[4] Farrar JJ (2019). Stopping the Gaps in Epidemic Preparedness. N Engl J Med..

[5] Balsari S, Kiang MV, Buckee CO (2021). Data in Crisis - Rethinking Disaster Preparedness in the United States. N Engl J Med..

[6] Huang C, Koppel R, McGreevey JD, Craven CK, Schreiber R (2020). Transitions from One Electronic Health Record to Another: Challenges, Pitfalls, and Recommendations. Appl Clin Inform..

[7] **Heckman J.** House lawmakers pan VA EHR as ‘bad investment’ with upcoming $39B cost estimate. Federal News Network. Published July 27, 2022. Accessed November 30, 2022. https://federalnewsnetwork.com/veterans-affairs/2022/07/house-lawmakers-pan-va-ehr-as-bad-investment-with-upcoming-39b-cost-estimate/

[8] **Rucci JM, Bosch NA, Peterson D, Walkey AJ**. Estimating the Impact of COVID-19 on Invasive Mechanical Ventilation Trends in the United States. *Ann Am Thorac Soc*. Published online July 20, 2022. 10.1513/AnnalsATS.202205-467RL 10.1513/AnnalsATS.202205-467RL PMC9743481 35857409

[9] Arabi YM, Azoulay E, Al-Dorzi HM (2021). How the COVID-19 pandemic will change the future of critical care. Intensive Care Med..

[10] COVIDSurg Collaborative (2021). Effect of COVID-19 pandemic lockdowns on planned cancer surgery for 15 tumour types in 61 countries: an international, prospective, cohort study. Lancet Oncol..

[11] Chen RC, Haynes K, Du S, Barron J, Katz AJ (2021). Association of Cancer Screening Deficit in the United States With the COVID-19 Pandemic. JAMA Oncol..

[12] Park S, Stimpson JP (2021). Trends in Self-reported Forgone Medical Care Among Medicare Beneficiaries During the COVID-19 Pandemic. JAMA Health Forum..

[13] Patel SY, Mehrotra A, Huskamp HA, Uscher-Pines L, Ganguli I, Barnett ML (2021). Trends in Outpatient Care Delivery and Telemedicine During the COVID-19 Pandemic in the US. JAMA Intern Med..

[14] Fischer SH, Uscher-Pines L, Roth E, Breslau J (2021). The Transition to Telehealth during the First Months of the COVID-19 Pandemic: Evidence from a National Sample of Patients. J Gen Intern Med..

[15] Warner MA (2020). Stop Doing Needless Things! Saving Healthcare Resources During COVID-19 and Beyond. J Gen Intern Med..

[16] Dzara K, Pusic M, Carlile N, Krupat E, Alexander EK (2022). Educational adaptation to clinical training during the COVID-19 pandemic: a process analysis. BMC Med Educ..

[17] Uthlaut B, Catalanotti J, Kisielewski M, McGarry K, Finn K (2022). Hazard pay for internal medicine resident physicians during the COVID-19 pandemic: A national survey of program directors. J Hosp Med..

[18] About us. Veterans Affairs. Published October 12, 2022. Accessed November 30, 2022. https://www.va.gov/spokane-health-care/about-us/

[19] Locations | VA Spokane health care. Veterans Affairs. Accessed November 30, 2022. https://www.va.gov/spokane-health-care/locations/

[20] Morena AL, Gaias LM, Larkin C (2022). Understanding the Role of Clinical Champions and Their Impact on Clinician Behavior Change: The Need for Causal Pathway Mechanisms. Front Health Serv..

[21] Elo S, Kyngäs H (2008). The qualitative content analysis process. J Adv Nurs..

[22] Halling S, Lilleleht E, Krycka KC, Sayre G (2020). Vital Researcher Conversations: Pivoting Past Impasses in Qualitative Research. Journal of Humanistic Psychology..

[23] Carayon P, Schoofs Hundt A, Karsh BT (2006). Work system design for patient safety: the SEIPS model. Qual Saf Health Care..

[24] Gudi SK, Tiwari KK (2020). Preparedness and Lessons Learned from the Novel Coronavirus Disease. Int J Occup Environ Med..

[25] Sundararaman T, Muraleedharan VR, Ranjan A (2021). Pandemic resilience and health systems preparedness: lessons from COVID-19 for the twenty-first century. J Soc Econ Dev..

[26] **Snair M, Singaravelu S, Wollek S**. *Future Planning for the Public Health Emergency Preparedness Enterprise: Lessons Learned from the COVID-19 Pandemic: Proceedings of a Workshop*. National Academies Press; 2022. 10.17226/26805 37552788

[27] Bell SA, Abir M, Choi H, Cooke C, Iwashyna T (2018). All-Cause Hospital Admissions Among Older Adults After a Natural Disaster. Ann Emerg Med..

[28] Sharmin A, Rahman MA, Ahmed S, Ali SM (2021). Addressing critical success factors for improving concurrent emergency management: lessons learned from the COVID-19 pandemic. Ann Oper Res.

[29] Simonovic SP, Kundzewicz ZW, Wright N (2021). Floods and the COVID-19 pandemic-A new double hazard problem. WIREs Water..

[30] **Jerolleman A, Laska S, Torres J**. Lessons from Co-Occurring Disasters: COVID-19 and Eight Hurricanes. Published online 2021. https://hazards.colorado.edu/quick-response-report/lessons-from-co-occurring-disasters

[31] Doan XV, Shaw D (2019). Resource allocation when planning for simultaneous disasters. European Journal of Operational Research..

[32] Chambers DA, Glasgow RE, Stange KC (2013). The dynamic sustainability framework: addressing the paradox of sustainment amid ongoing change. Implement Sci..

[33] Hartzband P, Groopman J (2020). Physician Burnout, Interrupted. N Engl J Med..

[34] West CP, Dyrbye LN, Erwin PJ, Shanafelt TD (2016). Interventions to prevent and reduce physician burnout: a systematic review and meta-analysis. Lancet..

[35] Han S, Shanafelt TD, Sinsky CA (2019). Estimating the Attributable Cost of Physician Burnout in the United States. Ann Intern Med..

[36] Panagioti M, Panagopoulou E, Bower P (2017). Controlled Interventions to Reduce Burnout in Physicians: A Systematic Review and Meta-analysis. JAMA Intern Med..

[37] Pollock A, Campbell P, Cheyne J (2020). Interventions to support the resilience and mental health of frontline health and social care professionals during and after a disease outbreak, epidemic or pandemic: a mixed methods systematic review. Cochrane Database Syst Rev..

[38] Hicks JM (2020). Leader Communication Styles and Organizational Health. Health Care Manag (Frederick)..

[39] Lo D, McKimm J, Till A (2018). Transformational leadership: is this still relevant to clinical leaders?. Br J Hosp Med (Lond)..

[40] CDC. COVID Data Tracker. Centers for Disease Control and Prevention. Published March 28, 2020. Accessed November 30, 2022. https://covid.cdc.gov/covid-data-tracker

